# ENGAGING METHOD AND ANALYSIS TO DISCOVER AND REIMAGINE POSSIBILITIES FOR INDIVIDUALS AGING WITH AND INTO DISABILITY

**DOI:** 10.1093/geroni/igac059.309

**Published:** 2022-12-20

**Authors:** Kelly Munly, Ted Ng, Lieke van Heumen

## Abstract

This symposium engages diverse methods and analyses to discover and reimagine possibilities for individuals aging with and into disability. The presentations advance an understanding of the diversity of service/care needs as well as of social capital, with exploration into assistive technologies, educational attainment, and assessment of successful aging. In the first presentation, the authors delineate key informant interview data identifying capacity-building needs in professional skills, organizational operations, service/care models, and public policies to effectively serve older adults aging with and into disability. The second presentation offers a content analysis of how researchers conceptualize, measure, and apply social capital to developmental disability throughout the life course, reviewing peer-reviewed articles from 5 disciplines, across more than 20 years. In the third presentation, using National Health and Aging Trends Study data, the authors investigate the longitudinal associations between disabilities, assistive technologies, and subjective well-being among older adults. In the fourth presentation, the author uses multivariate regression analysis to analyze National Health Interview Survey (NHIS) data, building on prior research analyzing the association between educational attainment and the perceived need for future help with activities of daily living (ADLs), often measuring disability in the literature. In the fifth and final presentation, the authors use the National Health & Aging Trends Study data to understand successful aging from the perspectives of those who experience disability, integrating subjective components of self-rated health and well-being, and providing insights on subjective perceptions on successful aging among older adults with disabilities.

